# Combination strategy for prognostication in patients undergoing post-resuscitation care after cardiac arrest

**DOI:** 10.1038/s41598-023-49345-1

**Published:** 2023-12-11

**Authors:** Jung Soo Park, Eun Young Kim, Yeonho You, Jin Hong Min, Wonjoon Jeong, Hong Joon Ahn, Yong Nam In, In Ho Lee, Jae Moon Kim, Changshin Kang

**Affiliations:** 1 Department of Emergency Medicine, Chungnam National University Hospital, 282 Munhwa-ro, Jung-gu, Daejeon, Republic of Korea; 2 Department of Emergency Medicine, College of Medicine, Chungnam National University, 282 Mokdong-ro, Jung-gu, Daejeon, Republic of Korea; 3 Department of Neurology, Chungnam National University Sejong Hospital, 20, Bodeum 7-ro, Sejong, Republic of Korea; 4 Department of Radiology, Chungnam National University Hospital, 282 Munhwa-ro, Jung-gu, Daejeon, Republic of Korea; 5 Department of Radiology, College of Medicine, Chungnam National University, 282 Mokdong-ro, Jung-gu, Daejeon, Republic of Korea; 6 Department of Neurology, College of Medicine, Chungnam National University, 282 Mokdong-ro, Jung-gu, Daejeon, Republic of Korea

**Keywords:** Outcomes research, Cardiology, Neurology

## Abstract

This study investigated the prognostic performance of combination strategies using a multimodal approach in patients treated after cardiac arrest. Prospectively collected registry data were used for this retrospective analysis. Poor outcome was defined as a cerebral performance category of 3–5 at 6 months. Predictors of poor outcome were absence of ocular reflexes (PR/CR) without confounding factors, a highly malignant pattern on the most recent electroencephalography, defined as suppressed background with or without periodic discharges and burst-suppression, high neuron-specific enolase (NSE) after 48 h, and diffuse injury on imaging studies (computed tomography or diffusion-weighted imaging [DWI]) at 72–96 h. The prognostic performances for poor outcomes were analyzed for sensitivity and specificity. A total of 130 patients were included in the analysis. Of these, 68 (52.3%) patients had poor outcomes. The best prognostic performance was observed with the combination of absent PR/CR, high NSE, and diffuse injury on DWI [91.2%, 95% confidence interval (CI) 80.7–97.1], whereas the combination strategy of all available predictors did not improve prognostic performance (87.8%, 95% CI 73.8–95.9). Combining three of the predictors may improve prognostic performance and be more efficient than adding all tests indiscriminately, given limited medical resources.

## Introduction

Cardiac arrest (CA) occurs annually in approximately 50–110 per 100,000 people worldwide^1^. A withdrawal of life-sustaining treatment (WLST) based on a predicted poor neurological outcome is the most common cause of death in patients undergoing post-resuscitation care after CA^2–4^. Therefore, in patients who are comatose after resuscitation from cardiac arrest, the prognostication should be performed to both inform patient’s relatives and to help clinicians to target treatments based on the patient’s chances of achieving a neurological recovery^5^.

Over the past decades, outcome prediction after CA has progressed towards a multimodal approach to ensure high accuracy, and the European Resuscitation Council (ERC) and the European Society of Intensive Care Medicine (ESICM) have recently published a prognostication strategy algorithm combining at least two abnormal predictors of any of six tests^5^. However, a major bias from self-fulfilling prophecy having a potential for WLST can affect this algorithm for prognostication in the patients with CA^5^. A special condition limiting the risk of self-fulfilling prophecy bias is the absence of an active WLST policy. This has been described in some studies conducted in countries or communities where treatment limitations are not accepted due to cultural, legal or religious reasons^6,7^.

Another limitation of the current prognostic algorithm is the insufficient evidence on how to combine predictors to effectively maximize prognostic accuracy^8^. There are six prognostic tests, and they cannot all be performed in clinical practice because not every facility has sufficient resources^9,10^. Therefore, finding and optimizing an effective combination strategy for prognostication given the limited medical resources in each facility is essential. Particularly, among the six prognostic tests, imaging techniques, such as computed tomography (CT) and magnetic resonance imaging (MRI), have shown high accuracy as prognostic tests in previous studies^11–13^; however, they are not readily available in all countries.

Therefore, the aims of this study were (1) to investigate the prognostic performance of post-CA care in an environment where WLST is infrequently performed, not only for single predictors but also for combination strategies, including a significant number of imaging studies, and (2) suggest an optimal combination strategy to improve prognostic performance with limited medical resources.

## Methods

### Study design and population

This was a single-center, retrospective, observational, registry-based study. We collected data from a tertiary-care hospital registry on patients with post-CA care after out-of-hospital cardiac arrest (OHCA). The requirement for informed consent was waived by the Institutional Review Board of Chungnam National University Hospital (CNUH IRB 2022-06-016) owing to the retrospective study design. Comatose adult patients (> 18 years old) treated with post-CA care after OHCA between May 2018 and June 2022 were included in this study. Among them, patients who underwent extracorporeal membrane oxygenation (ECMO) and only one prognostic test were excluded from this study.

### Post-cardiac arrest care

All patients received standard intensive care according to our institutional intensive care unit protocol based on the 2021 international guidelines for post-CA care^5^. All included patients underwent post-CA care, including targeted temperature management (TTM). TTM was performed using cooling devices (Arctic Sun^®^ 5000; BD, Franklin Lakes, NJ, USA). The target temperature was determined by the attending physician (33 vs. 36 ℃) according to hemodynamic status or CA characteristics, and then maintained for 24 h with rewarming to 37 °C at a rate of 0.25 ℃ per hour and monitored using an esophageal or bladder temperature probe. Midazolam (0.05 mg/kg intravenous bolus, followed by a titrated intravenous continuous infusion at a dose between 0.05 and 0.2 mg/kg/h) and paralytics (cisatracurium or rocuronium) were administered for sedation and to control shivering. If there was evidence of electrographic seizure or a clinical diagnosis of seizure, antiepileptic drugs, such as levetiracetam and/or valproate, were administered. The sedation level for all patients was assessed using a clinical sedation and agitation score [Richmond Agitation-Sedation Scale (RASS)]. Deep sedation, defined as a RASS score of 4 or 5, was maintained for the first 72 h after ROSC.

Since 2018, WLST has been authorized only under high restrictions in Korea, even if the family has a strong willingness to pursue WLST^14^. Legally, declarations of irreversible and unrecoverable status must be obtained from at least two physicians. Therefore, the physicians in charge of post-CA care do not encourage WLST, and it is restrictively performed in patients with brain death who are denied organ donation by the caregiver or family. In addition, Korea accepts a highly strict qualification for brain death, such as a flat activity (< 2 µV) in an electroencephalogram (EEG) for 30 min^15^.

### Data acquisition

#### Baseline characteristics

These variables were extracted from the data registry: age, sex, Charlson comorbidity index, sequential organ failure assessment score within the first 24 h after admission, witnessed collapse, bystander cardiopulmonary resuscitation (CPR), time from collapse to CPR (no flow time), time from CPR to the return of spontaneous circulation (ROSC; low flow time), first monitored rhythm, etiology of cardiac arrest, Glasgow Coma Scale (GCS) immediately after ROSC, time to the targeted temperature, and times to perform each prognostic test from ROSC. The following prognostic test data were extracted from the prospectively collected registry and electronic medical record system in our institution, Chungnam National University Hospital, Daejeon, Korea.

#### Ocular reflexes

Ocular reflexes included pupillary light and corneal reflexes (PR/CR), and an absent finding was defined that they were not observed bilaterally. Data for PR/CR were extracted between 72 and 96 h after ROSC and at the end of sedation to exclude confounding effects of sedative drugs. PR/CR measurements were performed by experienced nurses trained in general critical care, using a manual flash lamp and gauze.

#### Electroencephalography

EEG was performed for clinical indications at the discretion of the treating physicians in patients whose GCS motor score was below 6. The recordings were performed with the standard international 10–20 system of electrode placement of 21 electrodes for typically 15–30 min (Compumedics E-series, Compumedics, Melbourne, Australia). The EEG records were interpreted based on the recently updated guideline for critical patients by the American Clinical Neurophysiology Society^16^ and then qualitatively classified into one of three grades (benign, malignant, or highly malignant patterns) by two neurophysiologists (EYK and JMK) blinded to clinical course and outcome. Suppressed background, suppressed background with continuous periodic discharges, and burst-suppression background were defined as highly malignant patterns and used as poor outcome predictors. A consensus was made in the event of different interpretations of the EEGs between the two experts. Given the current guidelines and rarely performed WLST setting in this study, EEG recordings on the study population obtained between 24 h and 7 days from ROSC were identified through an EEG database. If more than one EEG was obtained, the last EEG of each patient was used for analysis.

#### Neuron-specific enolase

A peak value of serum neuron-specific enolase (NSE) measured at 48 or 72 h from ROSC was used for the analyses. All samples were obtained from an arterial line. Serum NSE levels were determined using an electrochemiluminescence immunoassay with Elecsys NSE^®^ (COBAS e801; Roche Diagnostics, Rotkreuz, Switzerland) in an authorized laboratory (GC Labs; Yongin, Geonggi-do, Korea).

#### Imaging studies

Brain CT and MRI, including diffusion-weighted imaging (DWI) performed after targeted temperature management (TTM) (72 to 96 h from ROSC), were used for this analysis. A gray-white matter ratio at basal ganglia level (GWR-BG) on CT and diffuse high-signal intensity (HSI) on DWI were calculated and estimated, respectively. Our protocol for the interpretation of imaging studies is described in the supplementary material (Supplementary Method and Supplementary Fig. S1).

### Outcome

Neurological outcomes were assessed at 6 months after OHCA using the Cerebral Performance Category (CPC) score (see Supplementary Note). The primary outcome in this study was a poor neurological outcome defined as CPC score of 3 to 5.

### Statistical analysis

Given that the individual numbers of each combination strategy are expected in this study, we chose a combination strategy with the smallest numbers as a more conservative estimate for sample size. Sample size was estimated using the method described by Buderer^17^. Based on a previous study using a Korean multi-center registry of post-cardiac arrest patients^18^, assuming that the combination strategy using the EEG and CT has a sensitivity of 30% and specificity of 100% for predicting poor neurological outcome in our cohort, a confidence interval (CI) of 20%, and a 80% prevalence of poor neurological outcome in a patients with post-cardiac arrest care, we calculated a sample size of at least 43 patients.

Categorical variables are presented as numbers with percentiles, and continuous variables are presented as means with standard deviation or median values with interquartile range, depending on the normality of the data. Categorical variables were compared between the groups using the Chi-square or Fisher’s exact test, as appropriate. Continuous variables were compared between groups using the Student’s t-test or the Mann–Whitney U-test. Receiver operating characteristic (ROC) curves were generated for each predictor, and a combined model was generated using logistic regression analysis. The predictive accuracy was determined by the area under the ROC curve (AUC), sensitivities, and false-positive rate (FPR) with 95% confidence interval (CI). Subsequently, the sensitivity was categorized and defined as excellent (> 90%), moderate (70 to 90%), or poor (< 70%)^19^. Inter-rater reliabilities were determined in the interpretations of EEG, CT, and DWI using Cohen’s kappa (κ) value and the intraclass correlation coefficient (ICC) according to the characteristics of the variables. The κ value was defined as slight (0.01 to 0.2), fair (0.21 to 0.40), moderate (0.41 to 0.60), substantial (0.61 to 0.80), and almost perfect (0.81 to 1.00) agreement^20^. An ICC value of < 0.4, 0.4–0.75, and > 0.75 indicated poor, fair to good, and excellent agreement, respectively^21^. Bootstrap internal validation was subsequently performed to verify the sensitivity and specificity for poor neurological outcome in each combination strategy. DeLong’s test was used to compare AUCs computed without multiple imputations. Statistical analysis was performed using IBM-SPSS 26.0 for Windows (IBM Corp., Armonk, NY, USA) and MedCalc 22.014 (MedCalc Software, Mariakerke, Belgium). The significance level was set at *P* < 0.05.

### Statement of ethics

The study was conducted according to the guidelines of the Declaration of Helsinki and approved by the Institutional Review Board (or Ethics Committee) of Chungnam National University Hospital (No. 2021-11-024). The extracted data included clinical data only; it includes no personally identifiable information. Therefore, the need for informed consent was waived.

## Results

### Study population

**Figure 1:**
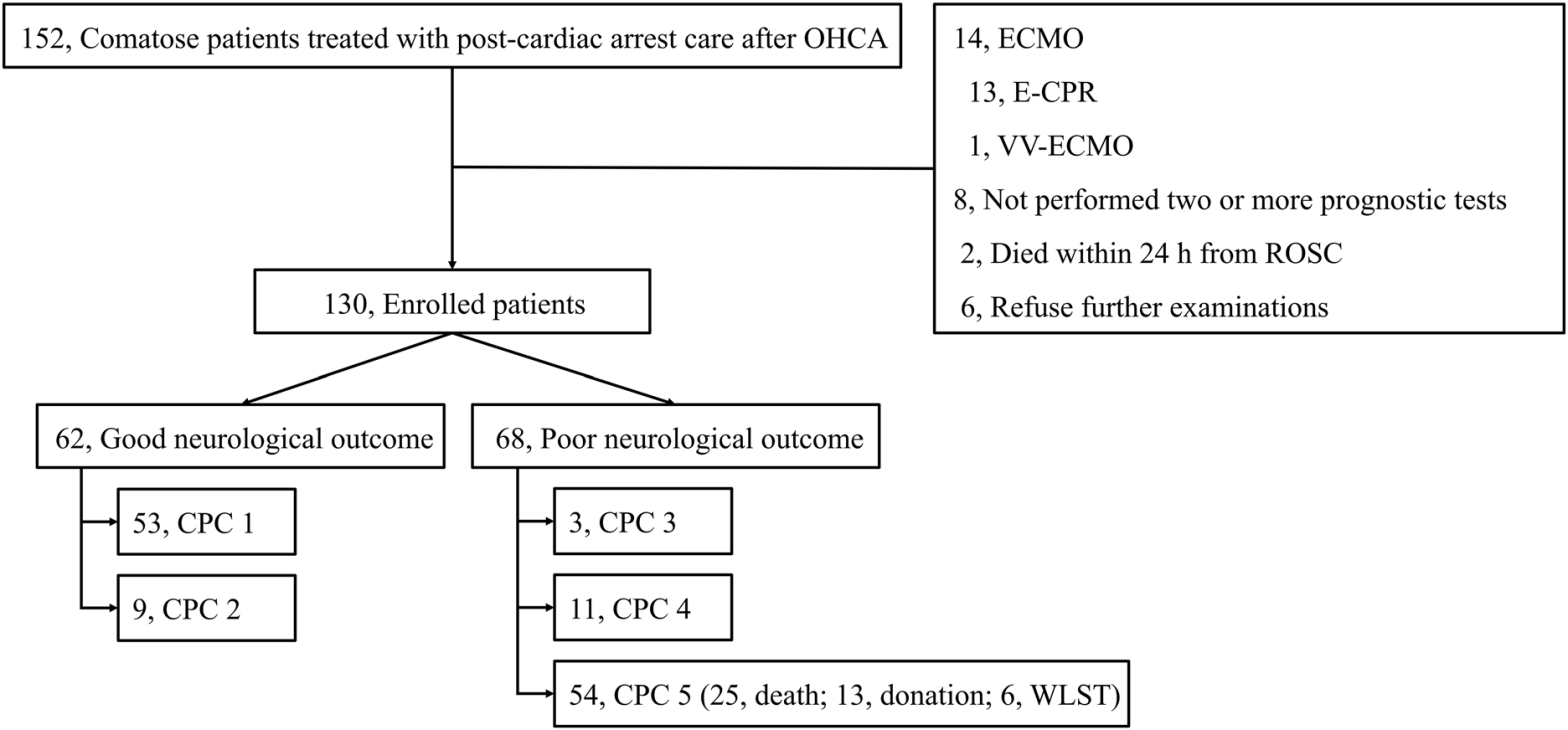
Flow diagram of included patients. *OHCA* out-of-hospital cardiac arrest, *ECMO* extracorporeal membrane oxygenation, *E-CPR* extracorporeal cardiopulmonary resuscitation, *VV-ECMO* venovenous extracorporeal membrane oxygenation, *ROSC* return of spontaneous circulation, *CPC* Cerebral Performance Category, *WLST* withdrawal of life-sustaining treatment.

Of the 152 patients who remained comatose state after post-CA care for OHCA, 14 patients who underwent ECMO (13, extracorporeal CPR; 1, venovenous ECMO for an acute respiratory distress syndrome), and 8 patients who did not receive at least two prognostic tests (6 refused additional prognostic tests due to medical cost; 2 died within 24 h from ROSC) were excluded from this study (Fig. 1). Of the 130 patients included, 62 (47.7%) and 68 (52.3%) showed good and poor neurological outcome, respectively. The distribution of CPC scores and cause of death after post-CA care are shown in Fig. 1.

The demographic and baseline characteristics of the study population according to the primary outcome are shown in Table 1. The number of patients who underwent each of the prognostic tests was 130 (100%) PR/CR, 72 (55.4%) EEG, 127 (97.7%) serum NSE, 74 (56.9%) CT, and 116 (89.2%) MRI. The times to perform EEG, CT, and MRI were not significantly different between patients with the good and poor neurological outcomes (Table 1).

**Table 1:** Baseline demographics and characteristics.

	Total patients, n = 130	Good neurological outcome, n = 62	Poor neurological outcome, n = 68	*p*
Age, years	57 (40–68)	57 (41–68)	57 (39–69)	0.84
Sex, male	96 (73.8)	51 (82.3)	45 (66.2)	0.05
Charlson comorbidity index	2 (0–4)	2 (1–4)	2 (0–4)	0.70
Cardiac arrest characteristics				
Witnessed, n	80 (61.5)	52 (83.9)	28 (41.2)	** < 0.001**
Bystander CPR, n	93 (71.5)	51 (82.3)	42 (61.8)	**0.01**
Shockable rhythm, n	43 (33.1)	37 (59.7)	6 (8.8)	** < 0.001**
No flow time, min	1.0 (10.0–30.0)	1.0 (0.0–2.3)	8.5 (0.0–22.3)	** < 0.001**
Low flow time, min	20.0 (10.0–30.0)	12.0 (8.0–19.5)	28.0 (19.3–37.0)	** < 0.001**
Arrest etiology, n				** < 0.001**
Cardiac	56 (23.8)	61 (66.1)	15 (22.1)	
Hypoxic	65 (50.0)	17 (27.4)	48 (70.6)	
Metabolic	6 (4.6)	2 (3.2)	4 (5.9)	
Anaphylactic	2 (1.5)	1 (1.6)	1 (1.5)	
Unknown	1 (0.8)	1 (1.6)	0	
SOFA score	10 (8–12)	9 (7–11)	11 (9–13)	0.06
GCS after ROSC	3 (3–3)	3 (3–5)	3 (3–3)	** < 0.001**
Targeted temperature, 33 ℃	113 (86.9)	50 (80.6)	63 (92.6)	0.07
Times, hours				
To targeted temperature	6.0 (4.6–7.5)	5.9 (4.5–7.4)	6 (4.6–7.9)	0.75
To EEG	96.5 (66.8–125.5)	94.4 (60.5–122.2)	96.5 (70.7–127.0)	0.43
To CT	76.2 (75.0–78.4)	75.8 (75.1–78.5)	76.9 (74.5–78.4)	0.68
To MRI	78.2 (76.2–79.9)	78.1 (75.8–79.4)	78.2 (76.5–80.3)	0.74

Data are presented as n (%) or median (interquartile range). p- values < 0.05 are marked bold.

*CPR* cardiopulmonary resuscitation, *SOFA* sequential organ failure assessment, *GCS* Glasgow Coma Scale, *ROSC* return of spontaneous circulation, *EEG* electroencephalogram, *CT* computed tomography, *MRI* magnetic resonance imaging.

### Prognostic performance of a single predictor

The prognostic values of each individual prognostic test are shown in Table 2. All poor outcome predictors—absent PR/CR, highly malignant EEG, high NSE, low GWR-BG, and diffuse HSI on DWI—were significantly associated with poor neurological outcome (Table 2). The AUC values, sensitivities, and FPRs of each individual predictor are shown in Table 3. Highly malignant EEG, high NSE greater than 144 ng/mL, and low GWR-BG less than 1.09 had 0% FPR with poor sensitivities of 62.5%, 52.3%, and 69.4%, respectively (Table 3). Additionally, absent PR/CR and diffuse HSI on DWI did not produce 0% FPR (Table 3).

**Table 2:** Associations between each prognostic tests and neurological outcomes.

Predictors	Measurement numbers			Findings	Total patients	Good neurological outcome	Poor neurological outcome	*p*
	Total	Good	Poor					
Clinical examination	130	62	68	Absent PR/CR	52 (40.0)	6 (9.7)	46 (67.6)	** < 0.001**
EEG	76	28	48	Highly malignant	30 (39.5)	0	30 (62.5)	** < 0.001**
Biomarker	127	62	65	Peak NSE, ng/mL	39.7 (21.1–176.0)	22.3 (16.7–30.6)	165.0 (65.9–292.9)	** < 0.001**
Imaging studies								
CT	74	38	36	GWR-BG	1.21 (1.06–1.27)	1.26 (1.22–1.33)	1.06 (0.96–1.19)	** < 0.001**
MRI	116	58	58	Diffuse HSI	57 (49.1)	1 (1.7)	56 (96.6)	** < 0.001**

Data are presented as n (%) or median (interquartile range).

p-values < 0.05 are marked bold.

*PR/CR* pupillary light and corneal reflexes, *EEG* electroencephalography, *NSE* neuron-specific enolase, *CT* computed tomography, *GWR-BG* gray and white matter ratio in basal ganglia level, *MRI* magnetic resonance imaging, *HSI* high-signal intensity.

**Table 3:** Prognostic performances of single predictors for poor neurological outcome.

Values	Cut-off	Sensitivity (95% CI)	Specificity (95% CI)	TP	FP	TN	FN
PR/CR (n = 130)	Absent	67.7 (55.2–78.5)	90.3 (80.1–96.4)	46	6	56	22
EEG (n = 76)	Highly malignant	62.5 (47.4–76.0)	100.0 (87.7–100.0)	30	0	28	18
NSE (n = 127)	144 ng/mL	52.3 (39.5–64.9)	100.0 (94.2–100.0)	34	0	62	31
GWR-BG (n = 74)	1.09	69.4 (51.9–83.7)	100.0 (90.7–100.0)	24	0	38	12
DWI (n = 116)	Diffuse HSI	96.5 (88.1–99.6)	98.3 (90.8–100.0)	49	1	48	2

Poor neurological outcome was defined as Cerebral Performance Category score of 3 to 5.

*CI* confidence interval, *TP* true positive, *FP* false positive, *TN* true negative, *FN* false negative, *PR/CR* pupillary light and corneal reflexes, *EEG* electroencephalography, *NSE* neuron-specific enolase, *GWR-BG* gray-white matter ratio at basal ganglia level, *DWI* diffusion-weighted imaging, *HSI* high signal intensity.

### Prognostic performance of combining strategies of multiple predictors

**Figure 2:**
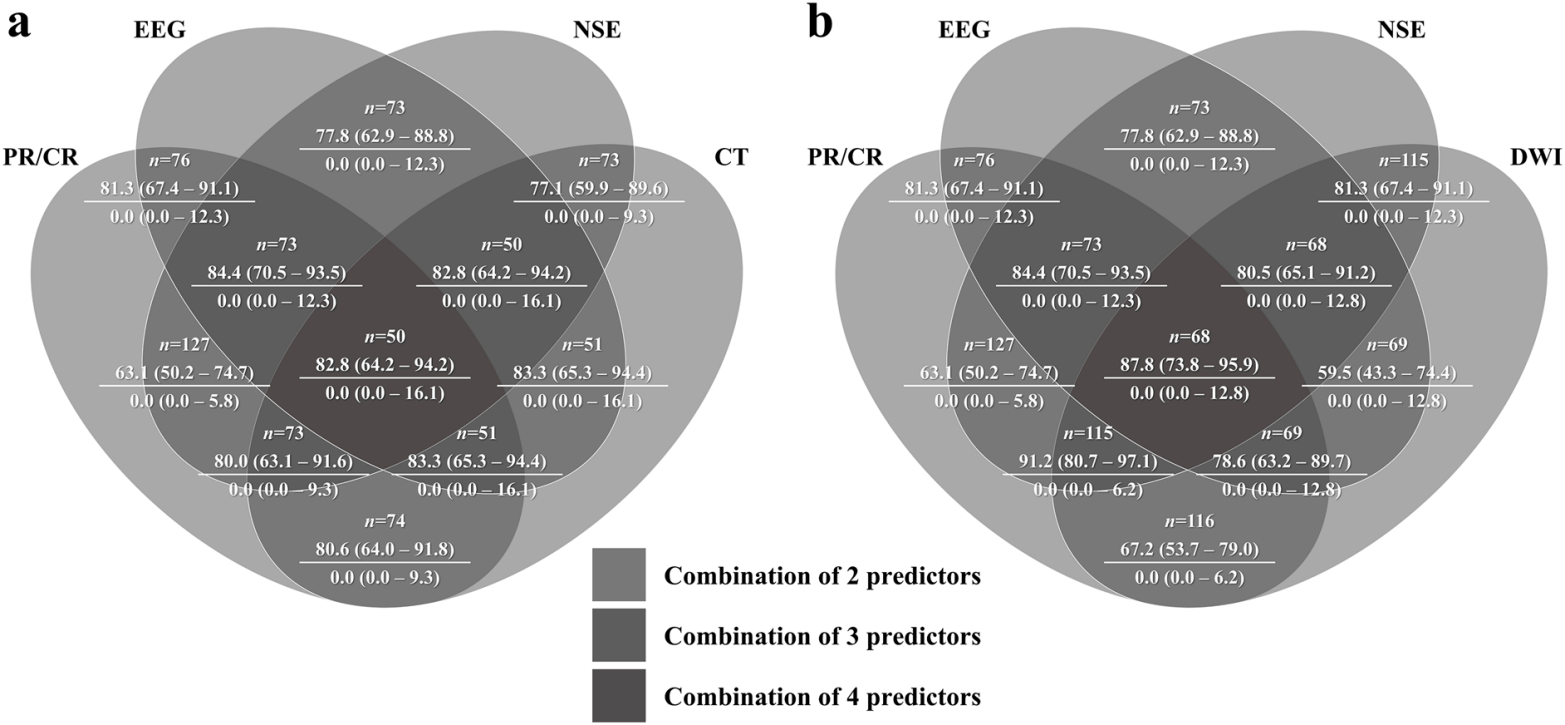
Venn diagrams showing the sensitivity (upper), FPR (below), and 95% CI of each combination strategy using 2 to 4 predictors: PR/CR, EEG, NSE, and/or (**a**) CT; (**b**) DWI. Asterisk: the areas is not correlated with the prognostic performance or the number of data. *CI* confidence interval, *CT* computed tomography, *DWI* diffusion-weighted imaging, *EEG* electroencephalography, *FPR* false-positive rate, *NSE* neuron-specific enolase, *PR/CR* pupil light and corneal reflexes.

The prognostic performance of combining strategies of two or more poor outcome predictors is shown in Fig. 2. All combination strategies produced 0% FPR for predicting poor neurological outcomes. Of the 18 combination strategies using multiple poor outcome predictors (the combination of two image studies was not performed), 3, 14, and 1 strategy(s) demonstrated poor (< 70%), moderate (70–90%), and excellent (> 90%) sensitivities, respectively, at 0% FPR (Fig. 2). All three combination strategies with poor sensitivity were observed in the combination strategies with two predictors (absent PR/CR + high NSE, absent PR/CR + diffuse HSI on DWI, and highly malignant EEG + diffuse HSI on DWI; Fig. 2), and the best prognostic performance was observed in the absence of PR/CR, high NSE, and diffuse HSI on DWI (91.2%, 95% CI 80.7–97.1, Fig. 2). The combination strategy of all available predictors (4 predictors, the others with one image study) rarely improved the prognostic performance (with CT: 92.8%, 95% CI 64.2–94.2; with DWI: 87.8%, 95% CI 73.8–95.9) compared with those in the combination strategies of three predictors (Fig. 2).

Imaging studies (CT and MRI) were used to verify the prognostic performance of the combination strategies, and showed no significant differences between the derivation and validation cohorts (Table 4).

**Table 4:** Internal validation of prognostic performance for the combination strategies using imaging studies.

Strategies	Derivation cohort, 94					Validation cohort, 36					^a^p
	AUC (95% CI)	Sensitivity (95% CI)	Specificity (95% CI)	NPV (95% CI)	PPV (95% CI)	AUC (95% CI)	Sensitivity (95% CI)	Specificity (95% CI)	NPV (95% CI)	PPV (95% CI)	
Combination strategies with CT											
PR/CR	0.96 (0.88–0.99)	85.7 (67.3–96.0)	100 (88.1–100)	87.9 (74.5–94.7)	100	0.71 (0.44–0.90)	62.5 (24.5–91.5)	100 (66.4–100)	75.0 (55.1–88.0)	100	0.10
EEG	0.98 (0.87–1.00)	90.9 (70.8–98.9)	100 (79.4–100)	88.9 (68.1–100)	100	0.70 (0.39–0.91)	62.5 (24.5–94.5)	100 (47.8–100)	62.5 (40.5–80.3)	100	0.09
NSE	0.97 (0.88–1.00)	81.5 (61.9–93.7)	100 (88.1–100)	85.3 (72.4–92.8)	100	0.78 (0.52–0.94)	62.5 (24.5–91.5)	100 (66.4–100)	75.0 (55.1–88.0)	100	0.14
PR/CR + EEG	0.98 (0.87–1.00)	90.9 (70.8–98.9)	100 (79.4–100)	88.9 (68.1–96.8)	100	0.70 (0.39–0.91)	62.5 (24.5–91.5)	100 (47.8–100)	62.5 (40.5–80.3)	100	0.09
PR/CR + NSE	0.97 (0.88–1.00)	85.2 (66.3–95.8)	100 (88.1–100)	87.9 (74.6–94.7)	100	0.72 (0.46–0.91)	62.5 (24.5–91.5)	100 (66.4–100)	75.0 (55.1–88.0)	100	0.10
EEG + NSE	0.99 (0.88–1.00)	90.5 (69.6–98.8)	100 (79.4–100)	88.9 (68.2–96.8)	100	0.78 (0.47–0.95)	62.5 (24.5–91.5)	100 (47.8–100)	62.5 (40.5–80.3)	100	0.13
PR/CR + EEG + NSE	0.98 (0.87–1.00)	90.5 (69.6–98.8)	100 (79.4 -100)	88.9 (68.2–96.8)	100	0.78 (0.47–0.95)	75.0 (34.9–96.8)	100 (47.8–100)	71.4 (42.9–89.3)	100	0.17
Combination strategies with MRI											
PR/CR	0.99 (0.93–1.00)	75.6 (60.5–87.1)	100 (91.8–100)	79.6 (70.0–86.7)	100	0.95 (0.80–0.99)	92.3 (64.0–99.8)	100 (78.2–100)	93.7 (69.5–99.0)	100	0.50
EEG	0.97 (0.88–1.00)	63.3 (43.9–80.1)	100 (82.4–100)	63.3 (51.9–73.4)	100	0.96 (0.76–1.00)	91.7 (61.5–99.8)	100 (63.1–100)	88.9 (55.1–98.1)	100	0.74
NSE	0.98 (0.93–1.00)	86.4 (72.6–94.8)	100 (91.8–100)	87.8 (77.3–93.8)	100	0.92 (0.78–0.99)	92.3 (64.0–99.8)	100 (78.2–100)	93.7 (69.5–99.0)	100	0.46
PR/CR + EEG	0.98 (0.89–1.00)	86.7 (69.3–96.2)	100 (82.4–100)	82.6 (65.6–92.2)	100	0.96 (0.76–1.00)	91.7 (61.5–99.8)	100 (63.1–100)	88.9 (55.1–98.1)	100	0.64
PR/CR + NSE	0.98 (0.93–1.00)	95.5 (84.5–99.4)	100 (91.8–100)	95.6 (84.7–98.8)	100	0.92 (0.76–0.99)	92.3 (64.0–99.8)	100 (78.2–100)	93.7 (69.5–99.0)	100	0.44
EEG + NSE	0.97 (0.88–1.00)	82.8 (64.2–94.2)	100 (82.4–100)	79.2 (63.1–89.4)	100	0.92 (0.71–0.99)	91.7 (61.5–99.8)	100 (63.1–100)	88.9 (55.1–98.1)	100	0.51
PR/CR + EEG + NSE	0.98 (0.89–1.00)	93.1 (77.2–99.2)	100 (82.4–100)	90.5 (71.4–97.3)	100	0.92 (0.71–0.99)	91.7 (61.5–99.8)	100 (63.1–100)	88.9 (55.1–98.1)	100	0.47

*AUC* area under the curve, *CI* confidence interval, *NPV* negative predictive value, *PPV* positive predictive value, *CT* computed tomography, *PR/CR* pupillary light and corneal reflexes, *EEG* electroencephalography, *NSE* neuron-specific enolase, *MRI* magnetic resonance imaging.

^a^p-value was calculated for the statistical comparison of prognostic performance between derivation and validation cohort by using DeLong’s test.

### Inter-rater reliability of EEG classification and imaging studies

The inter-rater reliabilities revealed moderate (κ = 0.489) and almost perfect (κ = 0.919) agreements in the interpretations of EEG and DWI, respectively (Table 5). GWR-BG showed a substantial agreement between the two reviewers (ICC, 0.808; Table 5).

**Table 5:** Inter-rater reliability analysis of interpretations for prognostic tests between two experts.

Values	Reviewer 1	Reviewer 2	Kappa or ICC value
EEG (n = 76)			
Highly malignant EEG, n (%)	23 (30.3%)	26 (34.2%)	0.489
CT (n = 74)			
GWR-BG, mean ± SD	1.15 ± 0.15	1.16 ± 0.16	0.808
DWI (n = 116)			
Diffuse HSI, n (%)	55 (47.4)	56 (48.3)	0.914

*ICC* intraclass correlation coefficient, *EEG* electroencephalography, *CT* computed tomography, *GWR-BG* gray-white matter ratio at basal ganglia level, *SD* standard deviation, *DWI* diffusion weighted imaging, *HSI* high-signal intensity.

## Discussion

This study found that the prognostic performance of combining prognostic strategies, regardless of included predictors, not only produced 0% FPR in combination but also generally revealed improved sensitivity compared with that of predictors that show poor sensitivity (< 70%) or unacceptable FPR individually. These findings emphasize the importance of multimodal outcome prediction to guarantee a low rate of falsely pessimistic predictions, potentially leading to an inappropriate WLST in patients undergoing post-CA care^5^. Nonetheless, the sensitivities of combinations of two predictors with 0% FPR were heterogenous from poor to excellent (> 90%). Combining 3 or 4 predictors demonstrated moderate to excellent sensitivities of 78.6% or more. Interpretations of poor outcomes from both imaging studies showed numerically higher inter-rater agreement compared with those of EEG (moderate in EEG vs. substantial to almost perfect in imaging studies).

Several previous studies reported that false positive findings occurred with all the single prognostic tests currently used for prognostication, emphasizing the importance of a multimodal approach^22–24^, and thus, combining predictors aims to increase the sensitivity of outcome prediction and reduce the risk of false, pessimistic prognostication. We found that the prognostic performance of a single prognostic test, of which two tests failed to achieve 0% FPR. The others not only had relatively low sensitivities (< 70%), but also a wide range of 95% CIs, again emphasizing the importance of a multimodal approach. We hypothesized that adding more predictors (i.e., maximal four predictors in this study due to limited medical resources and avoiding the combination of two imaging studies) to a combination strategy would be associated with improved accuracy in predicting poor neurological outcome. However, the strategies combining four predictors, regardless of the imaging study, did not show a significant improvement in sensitivity with 0% FPR compared with that of combination strategies using three predictors, which showed the best prognostic performance. Although the heterogeneous sample size for each predictor and the retrospective nature of this registry-based study are limitations, our findings indicate that imprudently adding predictors to the combined strategy without careful consideration does not guarantee the improvement of prognostic performance and may be inefficient when considering limited medical resources and costs. In line with this suggestion, a recent external validation study of the 2021 ERC/ESICM prognostic algorithm reported that the combination of prognostic tests representing complementary and/or duplicated pathophysiology, highly malignant EEG, and absent somatosensory evoked potentials (SSEP) response, or poor CT and poor MRI revealed even lower sensitivities compared with those of each of the individual predictors alone^18^. This supports that adding predictors, such as duplicated pathophysiology with another predictor, may increase redundancy rather than sensitivity^8^. Particularly in a resource-limited setting, utilizing and combining a few predictors should be maximized while still providing high accuracy for prognostication.

From our finding, an excellent sensitivity (> 90%) was observed solely in three predictors: absent PR/CR, high NSE, and diffuse HSI on DWI. The upper boundary of the 95% CI of the FPR was 6.2%, which is close to 5% and suggests a sufficient condition for the most robust predictors^25^. Additionally, internal validation tests showed that there were non-significant differences in the prognostic performance between each cohort (derivation vs. validation). These modalities represent individually different pathophysiologic mechanisms in hypoxic-ischemic brain injury^10,26,27^, these prognostic tests were measured or performed without major confounders. Notably, MRI was performed during the homogenous phase (i.e., post TTM period, 72 to 96 h after ROSC), whereas the EEG was not (i.e., between normothermia and post TTM period). Previous studies suggested that different prognostic tests all have optimal predictive value at specific time points after the arrest, requiring exact timing and organization of the prognostic diagnostic process to avoid suboptimal sensitivity and specificity of an individual test, and post-CA care should be taken to optimize the timing of the individual prognostic parameters to ensure optimal sensitivity and specificity^8,28^. However, a prospective multicenter evaluation or an external validation test with a large sample size in an unbiased and reproducible setting would allow the generalization of the combination strategy using the three predictors proposed in this study. Given the lack of evidence for this combination strategy, we suggest that these three predictors obtained in the post-TTM period likely have the potential to improve the prognostic performance by combining them.

Among the prognostic tests in the algorithm, five imaging studies can assist in prognostication after CA by visualizing injury patterns^29^. However, there is a dearth of data for imaging studies, particularly MRI studies, that have been used in studies on the multimodal approach to prognostication. For example, a study with large cohort of 585 patients enrolled only 35 (6.0%) patients’ MRI data^22^. In addition, there are no standardized recommendations as to definite times to perform, measurement techniques, and abnormal findings. Imaging methods, particularly MRI, have limitations for application such as the difficulty of moving patients out of the intensive care unit or a relatively long scan time^30^. Thus, it is reasonable to reserve the use of imaging studies for prognostication only in centers where specific experience is available^5^. There is still a lack of evidence for the prognostic value of imaging studies, and thus the current guideline does not propose specific criteria for “poor outcome likely” in imaging studies^5^. Nevertheless, imaging studies are not prone to interference from sedative drugs, and they can be assessed blindly^5^. Our finding that diffuse HSI on DWI did not yield 0% FPR as a single predictor follows prior reports^31,32^. Therefore, we suggest that DWI should be a complementary tool with other predictors, possibly for identifying different subtypes of hypoxic-ischemic brain injury after CA. The lowest sensitivity was observed for the combination of highly malignant EEG and diffuse HSI on DWI in this study. Additionally, the median time for EEG recording in this study was almost 100 h after ROSC during the heterogeneous phase (i.e., between normothermia and post-TTM period). Although this is in line with the current guideline recommendations for the timing of EEG^5^, it is still insufficient to exclude bias. EEG, which is widely available, is the most used prognostic tool after CA^33^. Several studies have demonstrated that early EEG (i.e., performed within 12 h after ROSC) has a significant prognostic performance for either good or poor neurological outcome^34,35^. Unfortunately, this study did not demonstrate the combination strategy used early EEG finding. The complementary role between EEG and MRI in comatose survivors of CA was described, and the authors explained the discordance between EEG and MRI by a different pathophysiological mechanism for their abnormal findings^36,37^. EEG is mainly sensitive to cortical brain damage, whereas MRI allows for easier identification of structural abnormalities of the neocortex, deep gray nuclei, or hippocampi in the cortical and subcortical gray matter^10^. Several studies found that patients with malignant EEG patterns do not reliably demonstrate MRI evidence of anatomic injury and suggested that performing MRI in patients with highly malignant EEG is unlikely to yield additional useful information for prognostication^36–38^. Based on those previous reports, our finding suggests that the low sensitivity of the combination of a highly malignant EEG and diffuse HSI on DWI supports a complementary role between EEG and MRI. Moreover, the combination strategy of using early EEG and MRI may be useful to know the prognosis of patients with CA.

The present study has several limitations to generalize for clinical practice. First, a major limitation was that all predictors suggested from the current guideline were not included for all patients (i.e., SSEP and status myoclonus were not included in this comparative analysis due to resource limitations), and this has limited the possibility to test different combinations to determine the optimal prognostic algorithm in this setting. Therefore, our study does not fully assess and compare the combination of prognostic strategies in the current ERC/ESICM prognostication strategy algorithm, which can lead to selection bias in this study^22^. Second, this was a single-center retrospective study with a relatively small sample size. This makes the validation test low quality and with limited generalization. Therefore, further multicenter prospective studies with larger sample sizes are required to enhance the generalizability of these findings. Third, although the prognostic tests,—PR/CR, NSE, and imaging studies—were performed or obtained in the homogeneous phase (i.e., post TTM period), the time to EEG was not well controlled since the indication for EEG was made at the discretion of the responsible physicians at various time points in this study. Fourth, the sedatives used in this study, especially midazolam, were administered during TTM and may have confounded the clinical examinations to assess PR/CR and potentially affect the EEG pattern. In this study, the examination for PR/CR was performed after TTM and without sedation; however, potential bias can produce a confounding effect from administered sedatives, which may affect these predictors (i.e., PR/CR and EEG). However, the most last (latest) EEG data, collected over 120 h after ROSC, were used in this analysis. Given the prolonged time for the prognostic test and the rarely performed WLST in this study, we suggest that this concern could be reduced despite the lack of evidence. Finally, we used a simple qualitative analysis of DWI, which may not be generally accepted. The quantitative analysis for MRI was suggested based on several studies^39–41^. However, it is rarely performed in clinical practice and is difficult to reproduce. Therefore, we suggest that a simple method using the extent of diffuse HSI on DWI could be more useful compared with previous complex methods when added as one predictor as part of a multimodal approach.

## Conclusion

The best prognostic performance was observed in the strategy combining three predictors: absent PR/CR, high NSE, and diffuse HSI on DWI. However, the combination of four predictors did not lead to improved prognostic performance. Therefore, thoughtlessly adding tests to a combination strategy may not guarantee the improvement of prognostic performance and may be inefficient, especially when considering limited medical resources and costs.

### Supplementary Information

Supplementary Information.

## Data Availability

The datasets generated and/or analysed during the current study are not publicly available due to their containing information that could compromise the privacy of research participants, but are available from the corresponding author on reasonable request.
