# Supplementary material for: Combination strategy for prognostication in patients undergoing post-resuscitation care after cardiac arrest
Source: Sci Rep. 2023 Dec 11;13:21880. doi: 10.1038/s41598-023-49345-1 (PMC10711008; doi:10.1038/s41598-023-49345-1)
Supplement: Supplementary file 1 — Supplementary Information. [file 41598_2023_49345_MOESM1_ESM.docx]

**Supplementary Information**

**Title:** **Combination strategy for prognostication in patients undergoing post-resuscitation care after cardiac arrest**

**Supplementary Method.** Computed tomography and magnetic resonance image protocols

**Supplemental Figure S1.**

**Supplementary Note.** Cerebral Performance Category score

**Supplementary Method.** Imaging studies

The brain CT was obtained in 5-mm slices using a 64-channel system (Somatom Sensation 64, Siemens Healthineers, Munich, Germany). Two board-certified neuroradiologists (IHL and DMK), who were blinded to clinical outcomes, measured the Hounsfield units (HU) of the putamen (P), caudate nucleus (CN), posterior limb of the internal capsule (PIC), and corpus callosum (CC) at the basal ganglia (BG) level. Circular regions of measurement (9–11 mm^2^) were manually placed over those anatomical regions, and the average attenuation in Hounsfield units was recorded. A grey-white matter ratio at basal ganglia level (GWR-BG) was calculated according to a previously reported equation: GWR-BG = (HU_P_ + HU_CN_) / (HU_PIC_ + HU_CC_) (Fig. 1). The averaged GWR-BG of the two reviewers was used in the analysis.

The brain MRI scanning was performed using a 3 T scanner (Achieva, Philips Healthcare, Amsterdam, The Netherlands) and included diffusion-weighted imaging (DWI), apparent diffusion coefficient measurements, and T2-weighted imaging. Forty continuous DWI sections per patient were acquired using the standard b = 1000 s/ mm^2^. As with CT, two neuroradiologists interpreted the presence or absence of diffuse high-signal intensity (HSI) on DWI without any information regarding the clinical course and outcome of the patient. Focal HSI on DWI suggesting a lacunar infarction was interpreted as absent diffuse HSI. If the two reviewers disagreed, the final interpretation was determined by a third reviewer, a stroke neurologist affiliated with another institution.

**Supplemental Figure S1.** Prognostic predictors in imaging studies: (A) a grey-white matter ratio at basal ganglia level on brain computed tomography; (B) normal finding on diffusion-weighted image (DWI); (C, D) diffuse high-signal intensities on DWI in cortex and cerebellum (red arrows) and deep grey matter (yellow arrows).


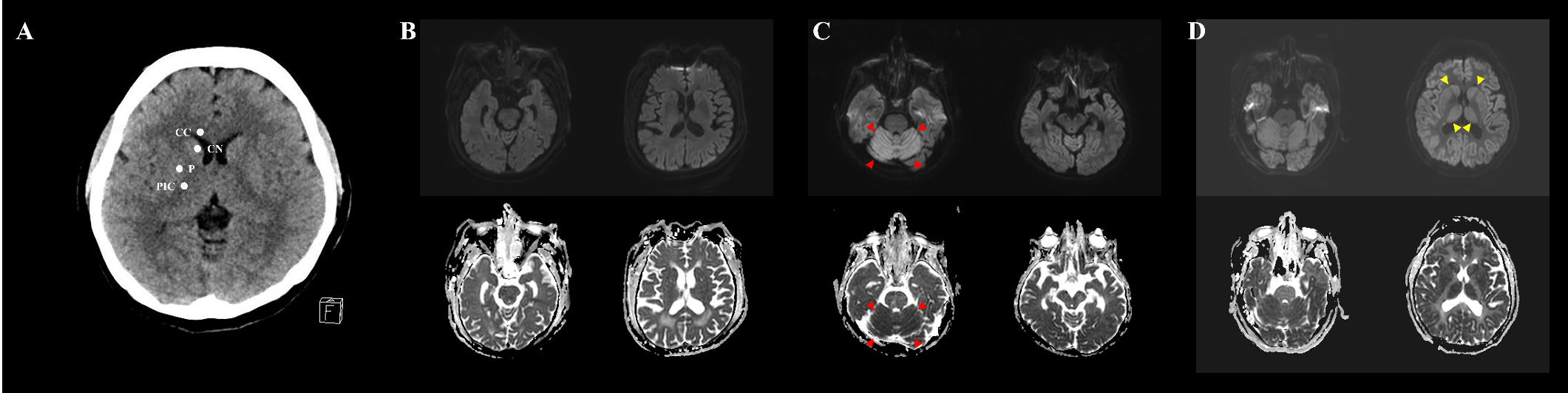


**Supplementary Note.** Cerebral Performance Category score

The Cerebral Performance Category (CPC) score ranges from one to five: 1, good cerebral performance or slight cerebral disability; 2, moderate disability, independence in activities of daily life; 3, severe disability, dependence on others for daily support; 4, coma or vegetative state; and 5, death or brain death. This was determined through either face-to-face or telephone interviews.
